# Combustion of a Solid Recovered Fuel (SRF) Produced from the Polymeric Fraction of Automotive Shredder Residue (ASR)

**DOI:** 10.3390/polym13213807

**Published:** 2021-11-03

**Authors:** Esther Acha, Alexander Lopez-Urionabarrenechea, Clara Delgado, Lander Martinez-Canibano, Borja Baltasar Perez-Martinez, Adriana Serras-Malillos, Blanca María Caballero, Lucía Unamunzaga, Elena Dosal, Noelia Montes, Jon Barrenetxea-Arando

**Affiliations:** 1 Department of Chemical and Environmental Engineering, Engineering Faculty of Bilbao, University of the Basque Country (UPV/EHU), 48940 Bilbao, Spain; alex.lopez@ehu.eus (A.L.-U.); landermartinez122@gmail.com (L.M.-C.); borjabperez@gmail.com (B.B.P.-M.); adriana.serras@ehu.eus (A.S.-M.); blancamaria.caballero@ehu.eus (B.M.C.); 2 AZTERLAN, Basque Research and Technology Alliance (BRTA), 48200 Durango, Spain; cdelgado@azterlan.es (C.D.); lunamunzaga@azterlan.es (L.U.); 3 Fundación Inatec, Environmental and Technological Innovation, 01170 Legutiano, Spain; edosal@fundacioninatec.es (E.D.); nmontes@fundacioninatec.es (N.M.); jbarrenetxea@fundacioninatec.es (J.B.-A.)

**Keywords:** combustion, automotive shredder residue, solid recovered fuel, alternative fuels, sustainable energy, waste-to-energy

## Abstract

The use of alternative fuels derived from residues in energy-intensive industries that rely on fossil fuels can cause considerable energy cost savings, but also significant environmental benefits by conserving non-renewable resources and reducing waste disposal. However, the switching from conventional to alternative fuels is challenging for industries, which require a sound understanding of the properties and combustion characteristics of the alternative fuel, in order to adequately adapt their industrial processes and equipment for its utilization. In this work, a solid recovered fuel (SRF) obtained from the polymeric fraction of an automotive shredder residue is tested for use as an alternative fuel for scrap preheating in an aluminium refinery. The material and chemical composition of the SRF has been extensively characterized using proximate and ultimate analyses, calorific values and thermal degradation studies. Considering the calorific value and the chlorine and mercury contents measured, the SRF can be designated as class code NCV 1; Cl 2; Hg 2 (EN ISO 21640:2021). The combustion of the SRF was studied in a laboratory-scale pilot plant, where the effects of temperature, flow, and an oxidizer were determined. The ash remaining after combustion, the collected liquid, and the generated gas phase were analysed in each test. It was observed that increasing the residence time of the gas at a high temperature allowed for a better combustion of the SRF. The oxidizer type was important for increasing the total combustion of the vapour compounds generated during the oxidation of the SRF and for avoiding uncontrolled combustion.

## 1. Introduction

In the European process industry, large amounts of energy and resources are used to produce millions of tonnes of materials every year. The use of scrap as a secondary raw material in metal-making processes reduces the depletion of natural resources. However, energy consumption remains a major concern. Although the smelting and refining of recycled scrap metal requires much lower energy inputs than those needed to produce primary metals from virgin mineral ores, electrical energy and fuel consumption still account for a large share of metal-making process costs. The implementation of scrap-preheating furnaces in the metal industry is a subject of interest due to the potential for economic and energy savings derived from the reduction in the melting time [1,2,3,4]. The developments in this field have so far been oriented towards the recovery of heat inherent in the off-gases generated during the melting process [3,5].

In the H2020 REVaMP project (Retrofitting Equipment for the Efficient Use of Variable Feedstock in Metal-making Processes), the innovation lies in the use of a polymeric waste stream as an alternative fuel for scrap preheating in an aluminium refinery (REFIAL, Otua Group, Spain). The goal is to reduce the overall fossil fuel consumption in the refinery, while minimising the amount of waste otherwise landfilled by other industries. Although energy costs and environmental concerns encourage cement companies worldwide to evaluate the extent to which conventional fuels can be replaced by waste materials [6], as well as the adequate equipment for their utilization, the use of waste-derived fuels (WDF) is not widespread in other process industries. In the aluminium industry, the possible co-combustion of seven types of WDF with propane was studied for STENA Metall’s aluminium recycling plant using combustion simulations provided by Ansys Chemkin-Pro software [7]. The study pinpointed the research needs related to pollutant formation and possible process changes induced by co-combustion. Two main challenges have been identified in the REVaMP project for the use of a WDF in the aluminium scrap preheater combustor: (1) to condition the polymeric fraction from the automotive shredder residue (ASR) to prepare a suitable Solid Recovered Fuel (SRF) in accordance with the requirements of the standard EN ISO 21640:2021 for the specifications and classes of solid recovered fuels; and (2) to design a preheating system for the scrap metal based on the characteristics of the aforementioned recovered fuel and the heating requirements of the aluminium scrap. Although the SRFs obtained from ASR are fuels with an appreciable energy content (18–36 MJ/kg) [8,9,10,11], their incineration in an industrial plant may have several limitations, due to the ash produced and due to the potential emissions of heavy metals, furans, and dioxins [12,13]. Therefore, the operating combustion conditions and the flue gas cleaning systems will have to be specifically adapted to allow the incineration of SRF, as laid down by the Industrial Emissions Directive 2010/75/EU (IED). Another difficulty to cope with regarding the combustion of SRFs arises from their heterogeneity, both with respect to particle form and size and to their composition. Several authors have highlighted the convenience of conducting further investigations into SRF combustion reactions at a pilot and industrial scale [8,9,14]. It is necessary to go beyond the standard laboratory methods developed specifically for SRF properties and the commonly used thermo-gravimetric analyses [8,10,15,16] so that larger quantities of SRF can be investigated at the original grain size.

The present paper deals with the research conducted to understand the combustion behaviour of SRFs prepared from ASR, in order to draw conclusions for the design, engineering and operation of the combustion chamber and of the heat exchanger of the scrap preheating system in the aluminium refinery, as well as to define the waste conditioning requirements to improve its fuel properties and to reduce its polluting, fouling and corrosion potential. An experimental study in a tank reactor was proposed with the aim of advancing the knowledge of the combustion performance of SRFs prepared from ASR, the composition of the combustion gases and condensate, and the solid residue (bottom ash) characteristics, which could supplement the analytical determination of the physical and chemical fuel properties.

## 2. Materials and Methods

### 2.1. ASR-Derived SRF Preparation and Characterization

The material employed in this study was the heavy fraction of ASR, a rejection stream generated during the treatment of end-of-life vehicles by a recycling company (DEYDESA, Otua Group, Spain), which specialises in the recovery of metals from complex solid waste.

The heavy ASR fraction is a very heterogeneous polymeric waste material, most of which is disposed of in industrial non-hazardous waste landfills. Only a small portion is used as fuel in energy production facilities (cement kilns). However, the net calorific value of this stream is high enough to be considered for the preparation of a solid recovered fuel for scrap preheating in the aluminium refinery of the Otua Group (REFIAL).

During SRF preparation, the heavy ASR fraction was ground in a cutter mill and sieved to a particle size in the range from 25–15 mm. The next treatment step was the removal of pieces of material with halogen content >1 wt.%, using X-Ray Transmission (XRT) technology in an automated sorting line (Figure S1). At this stage, almost 40 wt.% of the heavy fraction was rejected. The remainder, more than 60 wt.% of the input mass, was the solid recovered fuel (SRF) evaluated in this work.

The prepared SRF was characterized regarding its material and chemical composition. Representative samples were manually sorted into the categories of plastics, wood, textile, foam and others. The sorted materials were further analysed by Thermo Fisher Scientific portable analyser Niton™, X-Ray Fluorescence (XRF) (Waltham, MA, USA), in terms of metals content (Cr, Ba, Ti, Cl, Sb, Sn, Cd, Pb, Br, Zn, Cu, Ni, Fe, V, Bi, Se, As, Hg, and Au). Additionally, unsorted SRF samples were analysed by ICP-MS and GC-MS by an external laboratory for Sb, As, Cd, Co, Cu, Cr, Mn, Hg, Ni, Pb, Tl, V and PCBs, and for determination of halogen and sulphur containing oxygen (calorimetric bomb), and the subsequent specific titration analysis of the combustion product using different analytical techniques (EN 14582 for total Br, total S and total I; EN 1589 for total Cl, and EN 15408 for total F). These are chemical elements with threshold limits specified in their SRF acceptance criteria by local cement kilns, resulting from environmental regulations (IED) and operational requirements.

The SRF samples were also tested for their fuel properties (proximate analysis and calorific value) and underwent a preliminary thermal degradation study. A TA Instruments (New Castle, DE, USA) thermobalance SDT 650 with DSC/TGA was used to carry out the thermal decomposition study of the SRF. Two thermal degradation experiments were performed in air to measure the mass loss of SRF samples with time and temperature during a continuous heating process. Approximately 50 mg of SRF was loaded into an alumina crucible for each experiment. The temperature was increased from 25 °C to 950 °C at a heating rate of 10 °C/min, in air atmosphere. In addition, a 3 g SRF sample was heated in a Nabertherm (Lilienthal, Germany) LT5/11 muffle furnace with B410 controller and the loss on ignition (LOI) values were measured at various temperatures, by weighing the mass of the sample every 50 °C, from 250 °C to 850 °C, until a constant weight on a precision balance.

Additionally, proximate and ultimate analyses were performed on SRF samples milled to a particle size of approximately 1 mm by cryogenic grinding. The proximate analysis was carried out on the LECO TGA-700 (Stevensville, MI, USA) thermobalance, following the ASTM D7582 method. The analysis of the elements C, H, N, and S was carried out on LECO TrueSpec CHN and S automatic elemental analysers. For the elemental analysis of halogens (Cl and Br), the UNE-EN 15408 standard was followed, with a calorimetric pump LECO AC-500 and the analysis of the dissolved chlorides and bromides by DIONEX (Watertown, Massachussetts, USA) ICS-3000 ion chromatograph. Finally, the determination of the calorific value of the residue was carried out by measuring the Gross Calorific Value (GCV), on a dry basis (d), in the LECO AC-500 calorimetric pump and calculating the Net Calorific Value (NCV) as received (ar). In industrial practice, this was the maximum heat available in the combustion of this waste and is also one of the properties used to classify a solid recovered fuel (SRF) into class codes in accordance with the standard EN ISO 21640:2021.

### 2.2. Combustion Tests

Combustion tests were carried out in a laboratory scale pilot plant installation shown in the Supplementary Material (Figure S2). The installation was made up of a 2 L tank reactor, a 0.38 L packed-bed tubular reactor (0.6 m long), a condenser train, an active carbon adsorption step and a gases collection system. Controlled flow rate of air, oxygen or enriched air could be fed into the system. The two reactors were heated by electric furnaces equipped with heating controllers. The tubular reactor, which could be by-passed or connected in series to the outlet of the tank reactor, was packed with an inert solid filler (refractory brick ground to 1 mm particle size) to improve the heat transfer and fluid dynamics inside the reactor. A K-type thermocouple was used at the outlet of the tubular reactor to measure the flue gas temperature. The condenser train allowed for the fractional condensation of volatiles of the flue gas and was used to collect condensates for further analysis.

The tubular reactor was employed with two different purposes. In some experiments, it was used to increase the residence time of vapours and gases at the same temperature as set in the tank reactor. This provided time to complete the gas phase combustion reactions that were initiated in the tank reactor. Alternatively, in other experiments, it acted as a “post-combustion” chamber at 900 °C, to promote the thermal decomposition of organic compounds.

The combustion tests carried out, and the main conditions under which they were performed, are summarized in Table 1. A first set of experiments was performed to select the SRF/air ratios in the system and to assess the need for increasing residence time of evolved gases. The temperature of the combustion was selected, taking into consideration the thermal degradation of the SRF, which is presented later in Section 3.2. The objective of those first runs was to achieve the most complete combustion and stable reaction possible. After the setting-up phase, a second set of experiments (Tests 5–10) was executed, which was designed to evaluate combustion (plus post-combustion) at different oxidative atmospheres with increasing vol.% O_2_ (air, enriched air, and pure oxygen) and with two different temperatures in the tubular reactor. The highest temperature, 900 °C, was selected to reproduce the high temperature operating conditions of the post-combustors. For the combustion study, SRF sample grounded to 10 mm was employed.

The experimental procedure was as follows, for all the tests: 50 g of SRF was loaded at room temperature in the tank reactor. The tank reactor was heated up to 220 °C (100 °C/min rate) and, after reaching this value, the oxidizer supply started and the tank temperature was raised up to 550 °C with a 3 °C/min ramp and maintained at 550 °C until the end of the combustion process. The temperature of the tubular reactor, when employed, was set at 550 °C or at 900 °C. After each trial, the solid residue in the combustor (bottom ash) was collected and weighed. The residual carbon content in this solid was analysed three times and the uncertainties are provided in the results.

During the experiments, the colour of the combustion gases observed in situ was used as an indicator of combustion efficiency. Gases that were opaquer indicated that the combustion was not of a good quality, and more transparent gases were representative of a better combustion. The level of combustion in the tank reactor was later confirmed by analysing the carbon content in the ash using the LECO TrueSpec CHN analyser. The carbon content in the ash was limited to 3% according to IED directive.

In several combustion runs, real-time measurements of the concentrations of O_2_, CO_2_, CO, NO_2_, NO, NH_3_, SO_2_, VOC, HCl, and HF in the combustion gases were determined with a portable FTIR gas analyser, Gasmet DX-400, for stack emission testing, by connecting its sampling system to the outlet of the tubular reactor. In the tests run without online gas analysis, the amount of condensates was estimated by weight difference and their composition was determined afterwards. In the experiments with online gas analysis, the mass balance was closed by considering the remainder as combustion gases. In those experiments, the monitoring of the O_2_ concentration in the combustion gases helped to mark the end of the combustion.

For the interpretation of the results of the online composition analyses, detailed considerations are given here:

- The results are shown in concentration units and the combustion took place in a non-stationary state: the total gas volume varied during each test, as oxygen was consumed and combustion products were generated.
- The product concentration analysed at the process outlet at a given time resulted from a process in which the substances previously released from SRF (by thermal decomposition, partial or complete oxidation) were initially in contact with the oxidizer in the tank at the temperature reached at that time in the reactor and during the corresponding residence time, and, subsequently, in the tubular reactor at 550 °C or 900 °C during the residence time in this reactor.
- The gas analyser used was a standard stack emissions test equipment, calibrated to measure concentration of pollutants within typical emission ranges in the flue gases of industrial installations, after all the gas cleaning units. In the combustion tests presented in this work, the analyser measured the “raw combustion gases”. For some compounds, the concentration values were almost permanently above the calibration limits, but still within the measuring range of the analyser. Although exact concentration values could not be determined, important qualitative trend information could be obtained. The upper concentration limits of the analyser for the compounds, or groups of compounds, are given in the Supplementary Material (Table S2).
- When the concentration values were out of range for the instrument, no results were shown in the concentration vs. time plots.

## 3. Results and Discussion

### 3.1. Characterization of the Prepared SRF

**Figure 1:**
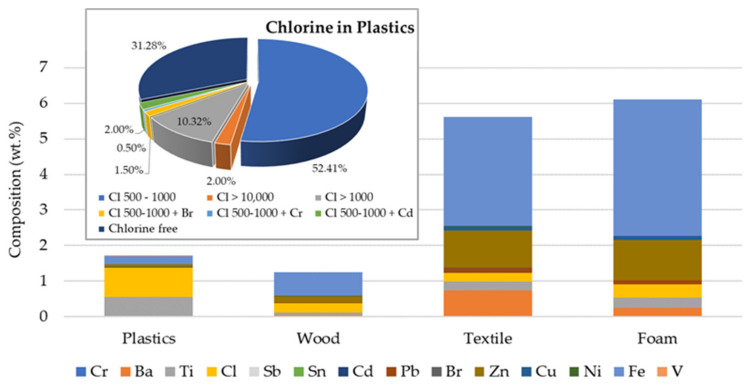
Results of elemental analysis of the main material fractions in the SRF, with breakdown of its chlorine content in plastics.

The SRF prepared is mainly composed of plastics (76.24 wt.%). The 5.30 wt.% of the SRF is wood, 1.36 wt.% is textiles, and foams account for only 0.64 wt.%. The remainder (16.46% of the total mass of the SRF) is mostly fines. The elemental composition of the four main material categories, as determined by XRF, is shown in Figure 1. Bi, Se, As, Hg, and Au were not detected in any of the four fractions. Chlorine, antimony, and iron are the most common elements in the plastics. Compared to the plastics stream, the chlorine content in the other material categories is very low, with iron as the prevailing element. In the case of wood, the elements detected in higher concentrations were iron, chlorine, and zinc. In the case of textile and foam materials, the main elements detected were Fe, Ba, Zn, Cl and Ti, with small amounts of Pb.

The presence of chlorine is clear in the plastics. While 31 wt.% of the total plastic mass in the SRF was free of chlorine, 56.4 wt.% of the plastics sampled had Cl contents in the range from 500–1000 ppm in weight. Chlorine contents above 1000 ppm were detected in 12.6 wt.% of the plastic fraction, and 2 wt.% of the plastic contained Cl > 10,000 ppm (1 wt.%). Overall, the average chlorine content determined in the SRF, according to the cement kiln test method, was 0.77 ± 0.19 wt.% for a total halogen (Br, Cl, F, I) content of 0.92 wt.%. The complete elemental analysis of the SRF is given in Table 2.

The proximate analysis of the SRF and the calorific value (higher and lower heating values) were also determined. The results obtained are shown below:

- Ash (550 °C) = 21.0 wt.% (LoQ = 0.001%);
- Dry matter (ar) = 97.9 wt.% (LoQ = 0.1%);
- GCV (d) = 27.9 MJ/kg (LoQ = 0.500 MJ/kg);
- NCV (d) = 26.3 MJ/kg (LoQ = 0.500 MJ/kg);
- NCV (ar) = 25.7 MJ/kg.

The net calorific value of 25.7 MJ/kg (ar) is in line with previous results for this waste stream in the Otua Group (historical GCV data of ASR fractions recovered in the industrial group show values of 24 MJ/kg for the heavy ASR fraction and 22 MJ/kg for the light fluff) and with the data reported in the literature.

Standard EN ISO 21640:2021 (superseding EN 15359:2011) specifies a classification system for solid recovered fuels and a template containing a list of characteristics for the specification of their properties, enabling the trade and use of SRFs supporting environmental protection. Considering the calorific value and the chlorine and mercury contents measured in the SRF characterization, it would be classified as SRF class code NCV 1; Cl 2; Hg 2 according to that standard.

The above results can be compared with those obtained in the proximate and ultimate analysis of the finely ground SRF samples (particle size = 1 mm), exhibited in Table 3. These results present low standard deviations, indicating that the fine milling preparation of the test samples was effective for homogenization. A low moisture content (1.5 wt.%) and ash content (13.3 wt.%) were determined. These values are significantly better, in terms of energy utilization, than those found in the literature for the shredder residues, where ash contents of more than 20 wt.% (up to 40 wt.% in some cases) are common [17,18,19]. They are also lower than the moisture and ash content determined on the 25 mm size sample, especially in the case of ash. The calorific value of the finer test sample was higher than that of the coarser SRF test sample. These discrepancies emphasize the heterogeneous nature of the SRF and how its characterization results can be affected by sampling and preparation methods [10].

Concerning elemental analysis, again the literature presents wide ranges for the three main elements (CHN): The C content varies between 20 and 70 wt.%, H between 3 and 8 wt.%, and N between 1 and 5 wt.% [17,18,19]. As can be seen in Table 3, the fine SRF sample analysed in this work presents a favourable combination of C, H, and N contents for combustion. That is, high values of C and H, which are the elements that mainly contain the chemical energy of the fuel; and low values of N, which, in addition to oxidizing endothermically, are an important source of some nitrogenous pollutants such as NH_3_ or NOx. As far as sulphur is concerned, the fine SRF analysed had a S content that was within the usual range for this element (0.2–1 wt.%) [17,18,19] and it did not differ greatly from the S content measured on the 25 mm sample (0.71 wt.%).

Regarding the halogen elements analysed, a total chlorine concentration of 1 wt.% was measured, which, although it was not as high as some values found in the literature for ASR (2–3 wt.%) [17,18], was high enough to generate corrosion and pollution problems derived from the generation of HCl, Cl_2_, dioxins, furans, and polychlorinated biphenyls. The threshold of 1 wt.% was set in this work for the SRF preparation, taking into account the existing acceptance limits for SRF in cement kilns and the regulatory requirements for waste (co-)incineration facilities set by the IED directive.

The amount of Br detected in the fine test sample was significantly lower than that of Cl (Table 3), and it was concluded that there appeared to be few brominated substances left in the analysed SRF samples and that chlorinated materials were the major concern. Similar results were obtained with the 25 mm sized SRF. The detected contents of Br, F, and I were one order of magnitude lower than those of Cl.

### 3.2. Thermal Analysis of SRF

The laboratory study of the thermal degradation of the SRF in air was performed through the thermogravimetric analysis (TGA) of microsamples. With a material as heterogeneous as SRF, the issue of the representativeness of the analysis samples was crucial. Several options exist to try to overcome this problem, as recommended in the EN/ISO standard methods for SRF characterization. The solution adopted in this study was to complement the laboratory TGA technique with less precise measurement methods that work with larger samples (determination of the LOI vs. T curve of 3 g samples).

**Figure 2:**
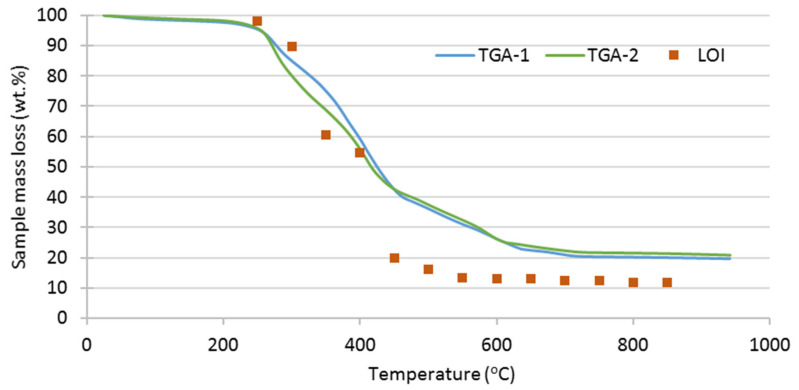
Compared thermal degradation of SRF (sample mass loss) in air and 10 °C/min obtained by thermogravimetric analysis and LOI measurement in a muffle furnace.

The TGA graphical results (Figure 2) indicate three different stages in the thermal decomposition of SRF, with different mass loss rates: the first in the interval around 250–450 °C, with the highest mass loss rate; the second, up to around 600 °C; and the third between 600 °C and 725 °C. From that temperature to the end of the measurements, a very slow mass loss is still observed, most likely while the chars formed are burning. These results are consistent with findings reported in the literature [4,6,7,20]. The results of LOI measurements at various ignition temperatures in the muffle (depicted also in Figure 2) shows a trend coincident with the TGA results, with major mass losses between 250 °C and 450 °C, extending to around 600 °C. The degradation ends at around 700 °C. This behaviour is different from that observed in SRF obtained in mechanical–biological treatment plants, where degradation increases up to more than 1300 °C [21].

### 3.3. Optimization of the Flow and Residence Time in Combustion Tests

Tests 1 to 5, described in Table 1, were used to select the combustion conditions and to establish the influence of the air flow rate and residence time of vapours and gases in the pilot plant. Based on the previous TGA and LOI analysis results, which indicated that most of the thermal degradation in air occurred below 600 °C, the temperature for the SRF combustion was set at 550 °C and the heating rates described in the Materials and Methods section were selected.

In solid waste combustions, above 40% of excess air is usually employed [22]. For that reason, Test 1 was carried out feeding 4.7 NL/min air (the stoichiometric flow was calculated to be 3.2 NL/min). The tubular reactor was not connected in series in this test. The employed air flow rate proved to be too high, making the residence time of the volatiles very low, and thus their combustion was very poor, as evidenced by the colour of the flue gas. As can be seen in Table 4, the ash remaining in the tank reactor after combustion complied with the maximum limit of 3 wt.% of organic carbon, set out in the Article 50 of the IED for slag and bottom ash formed in waste (co-)incineration plants. In this table, the amount of ash collected after each experiment is given (as a % by weight of the SRF sample fed), together with their C content. The pictures of the collected ashes are given in Table S1. The ash content of the combusted SRF was inside the wide range found in the literature, as revised by Mancini et al. [23].

In Test 2 a tubular reactor was placed after the tank reactor to increase the residence time of the volatiles, but no significant improvement was observed. Therefore, in Test 3, the air flow rate was decreased to stoichiometric to further increase the residence time. On this occasion, a less opaque flue gas was observed and the C content of the ash was kept below 3 wt.%. In view of this improvement, it was decided that the air flow rate should be lowered further (1.3 NL/min) in Test 4 to check whether the residence time was more critical for combustion quality than the oxygen availability. The appearance of flue gas continued to improve, but, in this case, the amount of air did not seem sufficient to burn the SRF completely, given that the carbon content of the ash was high (14 wt.% C, Table 4). Hence, an intermediate flow rate (2.2 NL/min) was chosen for Test 5. The C content in the ash, again below 3 wt.%, confirmed a sufficient combustion. Therefore, this oxidizer flow rate was selected for the rest of the experiments (Tests 6 to 10).

### 3.4. Combustion Tests with Online Analysis of Vapors

**Figure 3:**
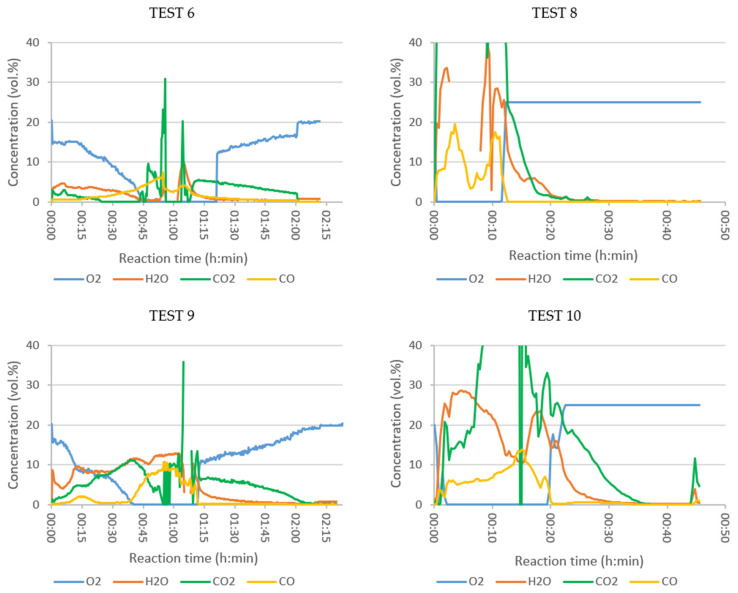
Concentration of O_2_, H_2_O, CO_2_, and CO (vol.%) in the flue gases of combustion of SRF: Test 6 (air, 550 °C), Test 8 (oxygen, 900 °C), Test 9 (air, 900 °C) and Test 10 (enriched air, 900 °C) (N.B.: CO_2_ curves are shown cut off at the top, as the *Y*-axis range is set from 0 to 40% to allow the proper visualization of the rest of the plotted values. The concentration curves plotted in the range 0–100 vol.% are shown in the graphs of Figure S5).

**Figure 4:**
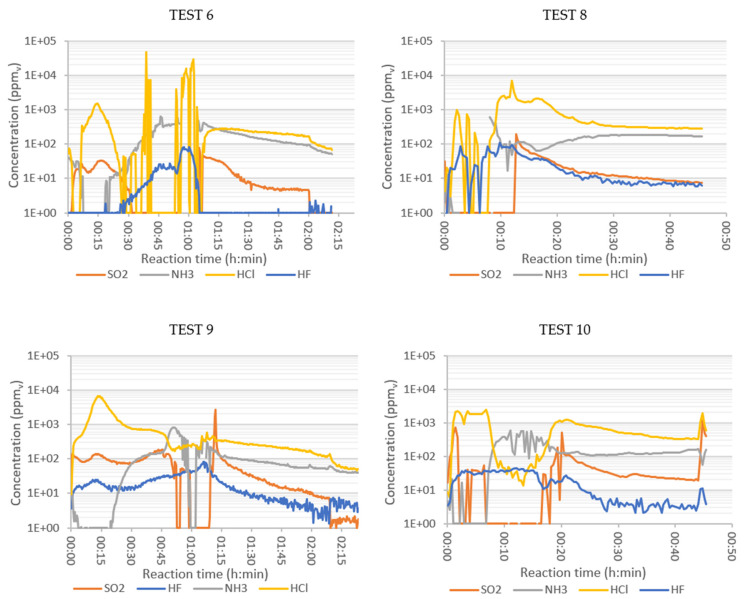
Concentration of SO_2_, NH_3_, HCl, and HF (ppm in volume) in the flue gases of combustion of SRF: Test 6 (air, 550 °C), Test 8 (oxygen, 900 °C), Test 9 (air, 900 °C), and Test 10 (enriched air, 900 °C).

**Figure 5:**
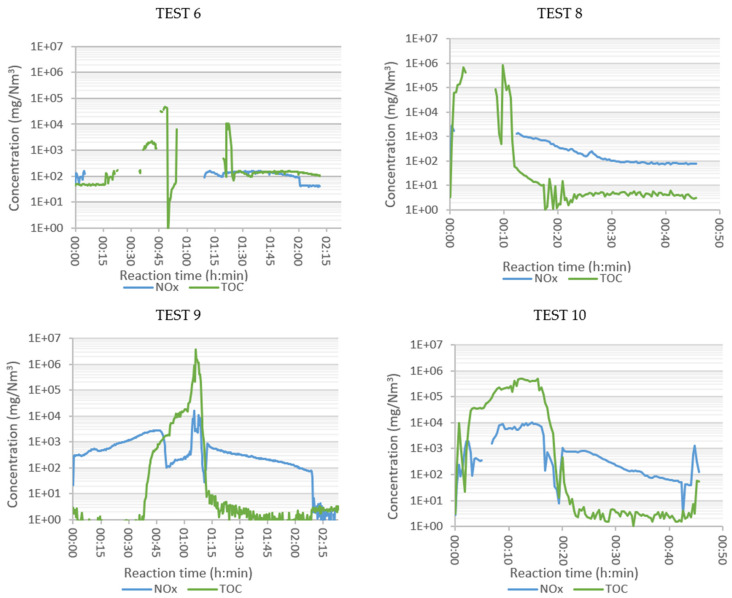
Concentration of Total Organic Compounds (TOC) and NOx (NO and NO_2_) in the flue gases of combustion of SRF: Test 6 (air, 550 °C), Test 8 (oxygen, 900 °C), Test 9 (air, 900 °C), and Test 10 (enriched air, 900 °C).

In Tests 6, 8, 9, and 10 the composition of the flue gas was analysed online, with the aim of examining the effect of the oxidizer employed and the temperature set in the tubular reactor on the SRF combustion process. The composition of the evolved gases analysed in each test, plotted as a function of time, are grouped in Figure 3, Figure 4 and Figure 5, arranged by families of compounds:

- Major compounds: O_2_, H_2_O, CO_2_ and CO (Figure 3);
- Specific pollutants: NH_3_, HCl, HF and SO_2_ (Figure 4);
- Grouped pollutants: TOC (representing all analysed organic compounds) and NOx (sum of NO and NO_2_) (Figure 5).

Oxygen concentration is the best indicator of combustion evolution. In Test 6 (Figure 3, air and T_tub_ = 550 °C), a slow but continuous oxygen consumption was observed during the first 45 min, and then remained at 0 vol.% for about 35 min. The maximum peaks of CO_2_, CO, and H_2_O were reached when all the fed O_2_ was consumed. In Test 10 (enriched air, T_tub_ = 900 °C) the CO_2_ production was higher, indicating better combustion, as also observed by Rey et al. when analysing the combustion of ASR [24]. The concentration of volatile organic compounds (Figure 5), grouped as TOC, increased rapidly in the period of maximum combustion (values out of range), indicating poor combustion.

The presence of ammonia was due to the existence of nitrogenous plastics in the initial sample, as observed in the elemental characterization of the SRF (Table 3). Ammonia is released from these polymeric chains and has the capacity to react with O_2_ to form N_2_ and H_2_O (and even nitrogen oxides). This would explain why the maximum ammonia production was detected when there was no oxygen at the outlet, i.e., when there was no oxygen available for its oxidation. The source of the NOx generated in Test 6 also seemed to be the nitrogenated groups contained in the polymeric residue, rather than the oxidation of N_2_ in the air, which required temperatures above 1090 °C in order to be oxidized [25].

HCl is the characteristic molecule of the thermal decomposition of organochlorinated substances. This compound begins to form at around 200 °C. This may explain the HCl peak observed for the early reaction times (Figure 4). In oxidative atmospheres it establishes an equilibrium reaction with Cl_2_, which also involves O_2_ as a reactant and H_2_O as a product [26]. The equilibrium of this reaction shifts to the left at elevated temperatures, i.e., there is more HCl than Cl_2_. The peak of HF concentration occurred in the period of maximum combustion, as in the case of NH_3_. Afterwards, its concentration decreased sharply to values close to zero. The oxidation of the sulphur present in the sample appeared to occur in two stages. During the first 30 min, two peaks of low concentration (below 40 mg/Nm^3^) appeared at temperatures below 260 °C. The highest concentrations were obtained at temperatures above 400 °C in the tank reactor, when combustion was more intense.

#### 3.4.1. Influence of the Tubular Reactor Temperature

Keeping the temperature of the tank reactor constant, but with the tubular reactor at 900 °C (Test 9), the combustion was accelerated. The oxygen concentration at the outlet decreased to zero about 5 min earlier than in Test 6 and started to recover almost 10 min earlier. This could be explained by the temperature effect on the kinetics of the combustion reactions occurring in the tubular reactor.

The combustion in Test 9 seemed to also be more efficient with the tubular reactor at 900 °C, as the production of CO_2_ and H_2_O was higher than in Test 6 from low reaction times, i.e., even at low temperatures in the tank reactor. In Test 9, the CO concentration did not start to increase until the depletion of oxygen in the flue gas; it decreased when O_2_ reappeared. It seemed that, in the central interval of the test, the massive generation of volatiles caused all the O_2_ to be consumed and was not available for CO oxidation to CO_2_.

The early peak of HCl, at low temperatures in the tank reactor, was significantly higher than the peak observed in Test 6. The 900 °C of the tubular reactor favoured the generation of HCl at the beginning of the thermal process. However, as the T_tank_ increased and the combustion progressed, the HCl concentration decreased. Regarding HF, a small peak was observed in the intense combustion interval, with similar maximum concentration values detected in Tests 6 and 9 (Figure 4).

The 900 °C in the tubular reactor resulted in a higher ammonia concentration in the flue gas after O_2_ depletion. The NOx concentration in Test 9 was higher than in Test 6. The SO_2_ concentration in Test 9 was much higher than in Test 6. This could be explained by the oxidation equilibrium of SO_2_ to SO_3_. This is an exothermic reaction, thermodynamically favoured at low temperatures, decreasing the SO_2_ concentration. This reaction would also explain the maximum SO_2_ concentration at a time when less O_2_ was available.

#### 3.4.2. Influence of Oxidizer

The effect of the oxidant was studied in Tests 8 (oxygen), 9 (air), and 10 (enriched air), all with the tubular reactor at 900 °C. The inlet flow in Tests 8 and 9 was 2.2 NL/min, but in Test 10 it was 3.5 NL/min (due to a limitation of the mass flow controllers). The residence time was, therefore, shorter in Test 10; on the other hand, the diffusion of oxygen in the air, which was necessary for combustion, was favoured. The heating of the tank reactor in the presence of air (Test 9) was constant and perfectly controlled up to 550 °C, reached in about 2 h. When enriched air was fed into the reactor (Test 10), the heating lasted approximately 35 min. However, when pure oxygen was injected (Test 8), the heating became out of control. Thermal runaway occurred, leading to a rapid and unstable reaction. At the end of this experiment the basket, in which the sample was loaded, was severely damaged (see Figure S4). The temperature variation in the tank reactor in Tests 8, 9 and 10 is shown in Figure S3.

When changing the comburent from air (Test 9) to pure oxygen (Test 8) the reactions started at lower temperatures and were much faster. The oxygen was completely consumed from the first moment and was recovered within 10 min. In Test 8, much higher concentrations of CO_2_ and H_2_O were observed at the point of maximum combustion, with a more complete combustion (higher CO_2_/CO ratio). In Test 10, with the excess of oxygen, the oxygen concentration in the flue gas quickly decreased to zero. The combustion consumed all of the oxygen, indicating that it would need more oxygen in this part. The CO_2_ concentration reached very high values, and the CO_2_/CO ratio indicated that C was oxidized to its maximum level.

The behaviour of NH_3_, HCl, HF, and SO_2_ with enriched air was, generally, between the behaviour observed in the tests with air and pure oxygen. The oxidant type affected the time when the maximum concentration of HF was detected but did not seem to affect the quantity. SO_2_ was not detected during the first 12 min in the combustion with oxygen (Test 8), most likely because it was depleted in other oxidation reactions. This would also be enhanced by the rightward shift of the exothermic equilibrium reaction, due to the lower temperature: SO_2_ + ½ O_2_ ←→ SO_3_. The HCl production in the flue gas did not seem to vary much with the oxidant, indicating that its concentration was more sensitive to temperature. Ammonia production was earlier in Test 8, associated with the acceleration of the combustion with pure oxygen. However, the maximum NH_3_ concentrations were detected in Tests 9 and 10.

As far as TOC is concerned, its presence is indicative of poor SRF combustion. In the pure oxygen test the combustion was uncontrolled, with many volatiles generated in a short time, high space velocity, short residence time, and with a lack of oxygen. The maximum NOx values detected were in the same range for the combustion in air and in enriched air. This seems to indicate that the NOx production does not depend so much on the type of oxidizer, but on the solid sample itself and its N content.

### 3.5. Liquid Analysis

The main composition of the condensate collected at the outlet of the tubular reactor is given in Table 5. The composition of the condensed liquid was measured only once, due to the small quantity collected. In the tests where online flue gas analysis was performed (Tests 6, 8, 9 and 10), the liquid was collected after the analyser. In the tests without the gas analysis, the flue gas leaving the tubular reactor was sent to a condensation system, where the gas phase was separated from the liquid phase.

The effect of the tubular reactor temperature on the flue gas is also clearly observed by comparing Test 6 (550 °C) and Test 9 (900 °C), both with air. The higher the temperature, the more water was present, indicating a better combustion of the SRF. When oxygen was used as a comburent (Tests 7 and 8), the water content was also high, but the instability of the combustion was reflected in the variability of the condensate composition. The optimum condensate composition was obtained with enriched air and a temperature of 900 °C in the tubular reactor, with almost 100% water (99.2 area %).

## 4. Conclusions

The general conclusions drawn from this work are described below. There is a special mention of the “3 Ts” of combustion: turbulence, time, and temperature. In the reaction system used, in which the residence time of the gases at a given temperature was small, the combustion was less complete at high air flow rates, even though this offered the advantage of operating closer to stoichiometric ratio of oxygen or even excess of oxygen. The minimum flow rate to be fed, in turn, was limited by the minimum turbulence that was guaranteed between the vapours generated and the oxidizer. It must be ensured that this minimum flow rate avoided the presence of unburned fuel in the ash.

The improvement in the combustion, resulting from the increase in the residence time and with the tubular reactor installed at the flue gas outlet of the tank reactor, was reinforced by increasing the temperature to 900 °C. Thus, for the minimum turbulence conditions, the residence time increased at a higher temperature. As a consequence, higher CO_2_ and H_2_O contents and a lower TOC was observed. At a higher temperature, the amount of water was greater in the condensed liquid and the number of organic compounds was smaller, indicating a better combustion of the SRF.

When modifying the comburent, it was observed that, with oxygen, more CO_2_ and H_2_O were produced, as well as many TOCs. The flow rate in this test was the same as the air flow rate, and thus the amount of unburned fuel was not due to bad turbulence. There were many combustion reactions requiring oxygen in a short time, so there was not enough oxygen in this period. Oxygen deficiency in the peak combustion period was also inherent to the discontinuous, non-stationary process. In the continuous process, already operating at a steady state, it is possible to feed the total amount of oxygen needed to avoid total oxygen consumption. Alternatively, a control loop can regulate the extra oxygen feed depending on the oxygen detected at the combustor outlet, to ensure that there is always enough oxygen inside the chamber.

A larger combustor would allow for a larger vapour/combustor contact volume, allowing for its operation at longer residence times to promote the complete and total oxidation of residues, even at high combustor flow rates. It is necessary to implement an afterburner stage, preferably at a high temperature and with an extra injection of comburent. Finally, it is important to note that operating with pure oxygen makes the combustion of the waste (exothermic reactions) very unstable and difficult to control.

## Figures and Tables

**Table 1:** Combustion tests performed. Reaction conditions with indication if the online gas analysis was performed.

Test	Oxidizer	Oxidizer Flow (NL/min)	Tubular Reactor T (°C)	Gases Analysis
1	Air	4.7	- ^2^	No
2	Air	4.7	550	No
3	Air	3.3	550	No
4	Air	1.3	550	No
5	Air	2.2	550	No
6	Air	2.2	550	Yes
7	Oxygen	2.2	900	No
8	Oxygen	2.2	900	Yes
9	Air	2.2	900	Yes
10	Enriched air	3.5 ^1^	900	Yes

^1^ 2.2 L/min of air mixed with 1.3 L/min of oxygen. ^2^ Tubular reactor was not employed in the first test.

**Table 2:** Characterization of the SRF according to cement kiln acceptance criteria.

Parameter	Value	Unit	Uncertainty (%)
Flash point	>150	°C	30
Sb	0.44·10^−2^	wt.%	32
As	0.06·10^−2^	wt.%	24
Pb	5.16·10^−2^	wt.%	32
Cr	0.85·10^−2^	wt.%	39
Co	0.15·10^−2^	wt.%	25
Cu	3.39·10^−2^	wt.%	28
Mn	2.72·10^−2^	wt.%	39
Ni	0.60·10^−2^	wt.%	30
V	0.10·10^−2^	wt.%	31
Hg	0.01·10^−2^	wt.%	28
Cd	0.07·10^−2^	wt.%	27
Tl	<0.5 10^−4^	wt.%	31
Hg + Cd + Tl	0.08·10^−2^	wt.%	31
Sb + As + Pb + Cr + Co + Cu + Mn + Ni + V	13.47·10^−2^	wt.%	39
total S	0.71	wt.%	20
total Br	0.06	wt.%	33
total Cl *	0.77	wt.%	23
total F	0.07	wt.%	29
total I	0.02	wt.%	50
total halogen	0.92	wt.%	50
PCBs (sum max. 7 compounds)	0.565	mg/kg	26

* mean value of 6 samples.

**Table 3:** Characterization of fuel properties of the fine SRF.

Proximate and Elemental Analysis (As Received, wt.%)			
Moisture	1.5 ± 0.1		
Organic content	85.2 ± 0.8 79.8 volatile material 5.4 fixed carbon ^2^	C	65.2 ± 1.0
		H	8.1 ± 0.2
		N	1.5 ± 0.3
		O	4.8 ± 1.0
		S	0.5 ± 0.1
		Cl	1.0 ± 0.2
		Br ^1^	2.44·10^−2^ ± 1.34·10^−2^
		Others ^2^	≈4.1
Ashes	13.3 ± 0.9		
**Calorific Value (MJ/kg)**			
GCV (dry)		30.8 ± 0.1	
NCV (dry)		28.9 ± 0.1	
NCV (as received)		28.4 ± 0.1	

^1^ In ppm. ^2^ By difference.

**Table 4:** Amount of ash and C content in ash obtained in the SRF combustion tests.

Test	Ash (wt.% SRF)	C Content (wt.% ash)
1	18.6	2.9 ± 0.3
2	26.0	2.8 ± 0.1
3	15.0	2.8 ± 0.4
4	14.0	14.2 ± 2.1
5	19.0	2.5 ± 0.2
6	34.0	7.6 ± 1.3
7	30.0	2.1 ± 0.2
8	24.0	2.2 ± 0.4
9	14.0	3.0 ± 0.1
10	20.0	2.2 ± 0.3

**Table 5:** Main composition of the liquid collected in the combustion tests of SRF.

Test	Water (Area %)	Other Organic Compounds (Area %)
1	48.7	43.2
2	50.4	45.3
3	81.5	12.9
4	77.9	19.5
5	n.c. *	n.c. *
6	36.1	58.4
7	69.1	11.5
8	92.2	7.8
9	68.8	7.4
10	99.2	0.8

* n.c.: not collected (liquids were not generated in the test).

## Data Availability

The data availability statement will be provided during the review process.

## Supplementary Materials

The following are available online at https://www.mdpi.com/article/10.3390/polym13213807/s1. Figure S1: SRF prepared from the heavy fraction of ASR: (a) output of the XRT sorting line; (b) test sample, Figure S2: Process flow diagram of the SRF combustion pilot plant, Figure S3: Variation of temperature recorded in the tank reactor: Test 8 feeding oxygen, Test 9 feeding air and Test 10 feeding enriched air, Figure S4: Condition of the basket in which the sample was placed after Test 8 feeding pure oxygen, Figure S5: Concentration of O_2_, H_2_O, CO_2_ and CO (vol.%) in the flue gases of combustion of SRF: Test 6 (air, 550 °C), Test 8 (oxygen, 900 °C), Test 9 (air, 900 °C) and Test 10 (enriched air, 900 °C), Table S1: Pictures of the collected ashes in the combustion tests. Table S2: Upper calibration limits of the equipment used in the online composition analysis of the vapours.
