# Supplementary material for: Combustion of a Solid Recovered Fuel (SRF) Produced from the Polymeric Fraction of Automotive Shredder Residue (ASR)
Source: Polymers (Basel). 2021 Nov 3;13(21):3807. doi: 10.3390/polym13213807 (PMC8587669; doi:10.3390/polym13213807)
Supplement: Supplementary file 1 [file polymers-13-03807-s001.zip › polymers-1341696-supplementary.pdf]

polymers

MDPI

Supplementary Material: Combustion of a Solid Recovered Fuel (SRF) Produced from the Polymeric Fraction of Automotive Shredder Residue (ASR)

Esther Acha, Alexander Lopez-Urionabarrenechea, Clara Delgado, Lander Martinez-Canibano, Borja Baltasar Perez-Martinez, Adriana Serras-Malillos, Blanca María Caballero, Lucía Unamunzaga, Elena Dosal, Noelia Montes, Jon Barrenetxea-Arando

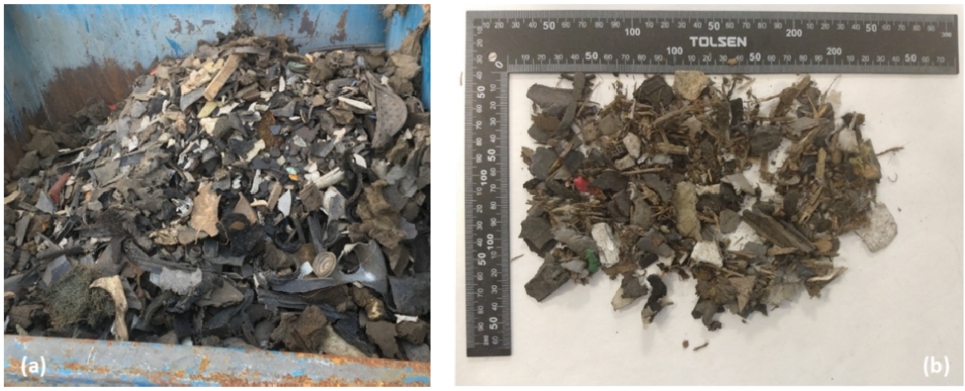

Figure S1. SRF prepared from the heavy fraction of ASR: (**a**) output of the XRT sorting line; (**b**) test sample.

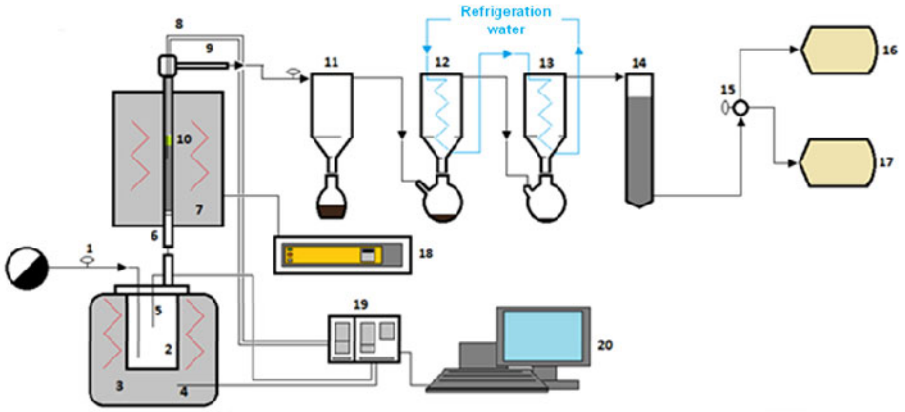

Figure S2. Process flow diagram of the SRF combustion pilot-plant.

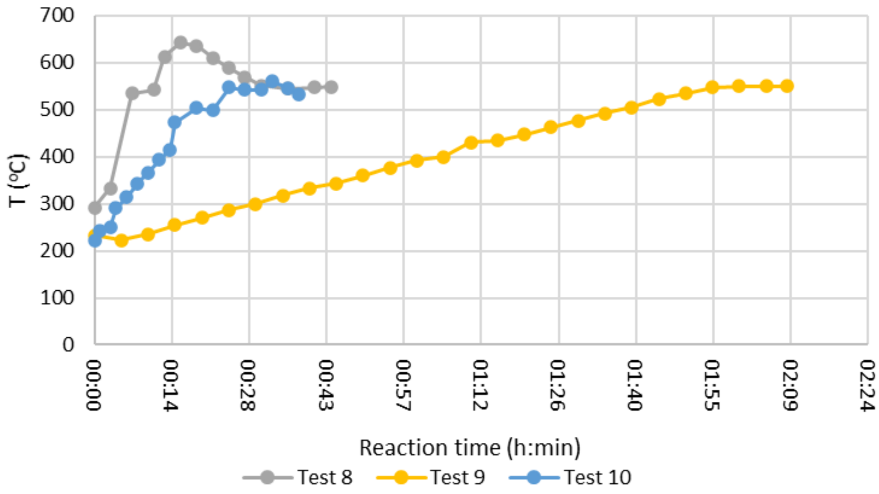

Figure S3. Variation of temperature recorded in the tank-reactor: Test 8 feeding oxygen, Test 9 feeding air and Test 10 feeding enriched air.

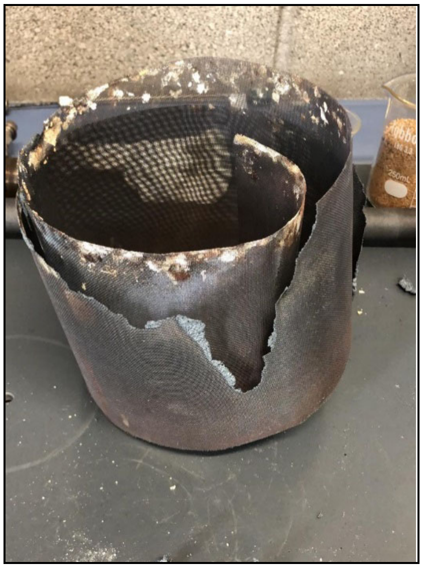

Figure S4. Condition of the basket in which the sample was placed after Test 8 feeding pure oxygen.

Table S1. Pictures of the collected ashes in the combustion tests.

TEST 1 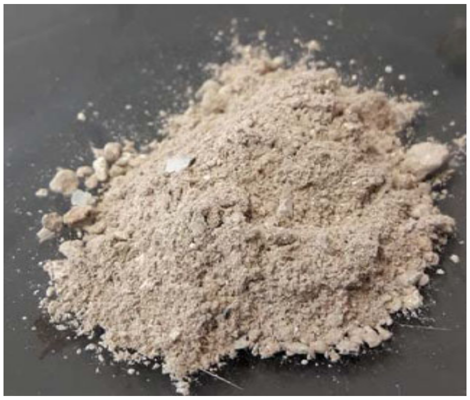	TEST 2 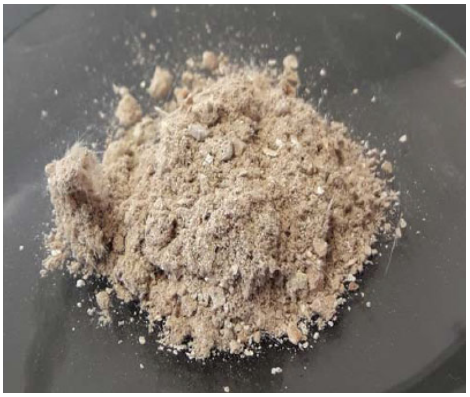
TEST 3 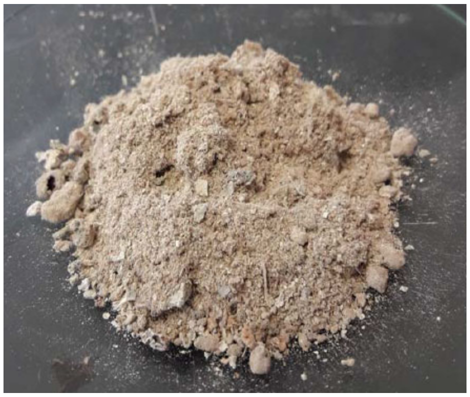	TEST 4 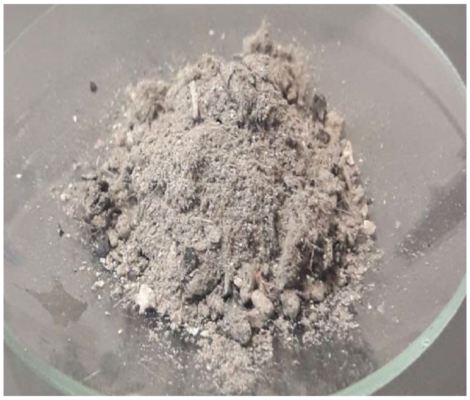
TEST 5 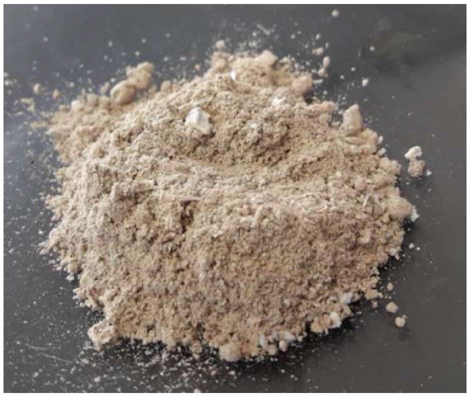	TEST 6 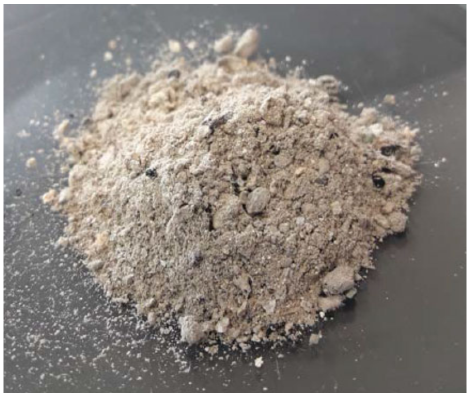

TEST 7

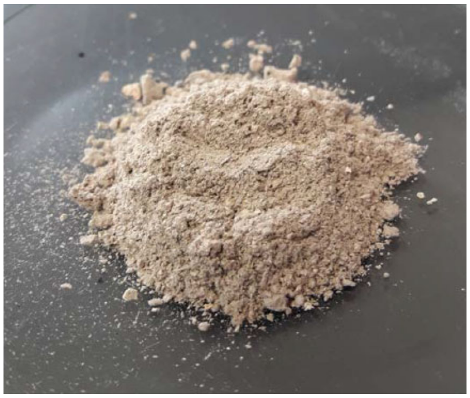

TEST 8

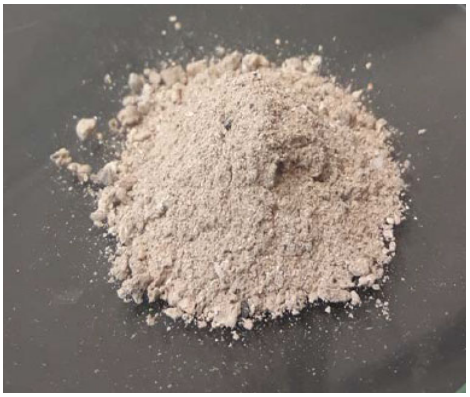

TEST 9

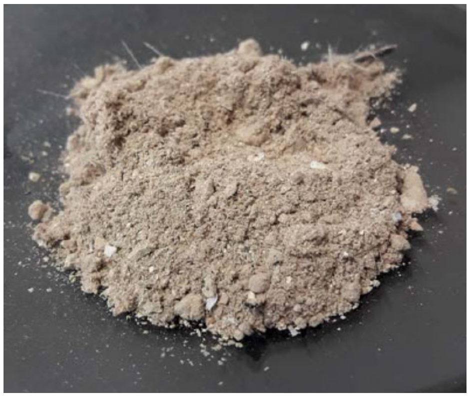

TEST 10

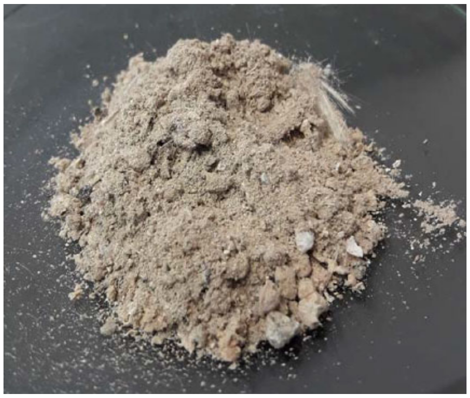

Table S2. Upper calibration limits of the equipment used in the online composition analysis of the vapours.

Compound	Upper calibration limit	
O_2	25	vol.%
H_2O	25	vol.%
CO_2	10	vol.%
CO	2	vol.%
SO_2	200	ppm_v
NH_3	20	ppm_v
HCl	10	ppm_v
HCl	10	ppm_v
HF	17	ppm_v
NOx	200	mg/Nm^3
TOC	50	mg/Nm^3

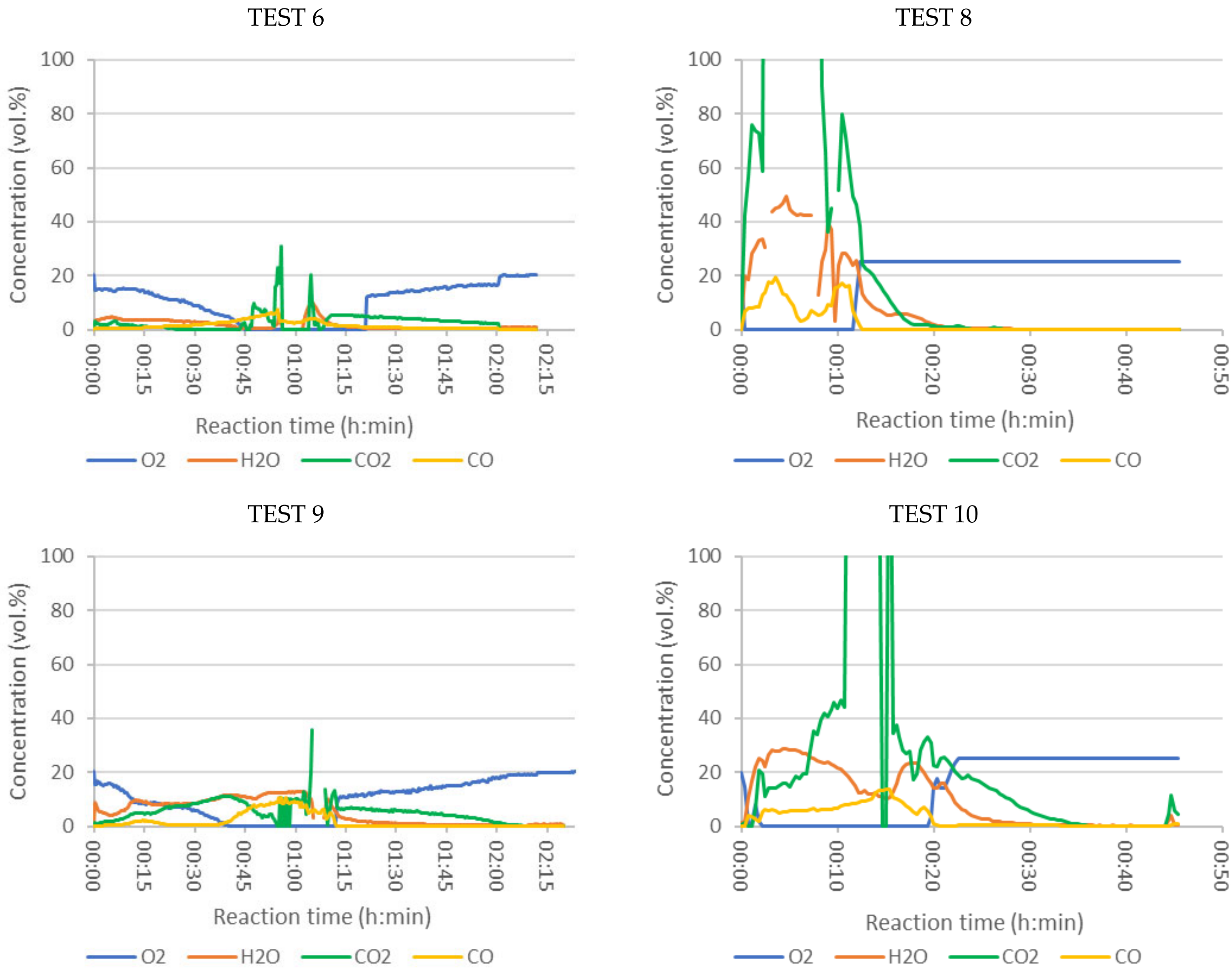

Figure S5. Concentration of O_2, H_2O, CO_2 and CO (vol.%) in the flue gases of combustion of SRF: Test 6 (air, 550 °C), Test 8 (oxygen, 900 °C), Test 9 (air, 900 °C) and Test 10 (enriched air, 900 °C).
